# Effect of a mixture of micronutrients, but not of bovine colostrum concentrate, on immune function parameters in healthy volunteers: a randomized placebo-controlled study

**DOI:** 10.1186/1475-2891-5-28

**Published:** 2006-11-21

**Authors:** Danielle AW Wolvers, Wendy MR van Herpen-Broekmans, Margot HGM Logman, Reggy PJ van der Wielen, Ruud Albers

**Affiliations:** 1 Unilever Food & Health Research Institute, Olivier van Noortlaan 120, NL-3133 AT, Vlaardingen, The Netherlands

## Abstract

**Background:**

Supplementation of nutritional deficiencies helps to improve immune function and resistance to infections in malnourished subjects. However, the suggested benefits of dietary supplementation for immune function in healthy well nourished subjects is less clear. Among the food constituents frequently associated with beneficial effects on immune function are micronutrients such as vitamin C, vitamin E, β-carotene and zinc, and colostrum. This study was designed to investigate the effects these ingredients on immune function markers in healthy volunteers.

**Methods:**

In a double-blind, randomized, parallel, 2*2, placebo-controlled intervention study one hundred thirty-eight healthy volunteers aged 40–80 y (average 57 ± 10 y) received one of the following treatments: (1) bovine colostrum concentrate 1.2 g/d (equivalent to ~500 mg/d immunoglobulins), (2) micronutrient mix of 288 mg vitamin E, 375 mg vitamin C, 12 mg β-carotene and 15 mg zinc/day, (3) combination of colostrum and micronutrient mix, or (4) placebo. Several immune function parameters were assessed after 6 and 10 weeks. Data were analyzed by analysis of variance. Groups were combined to test micronutrient treatment versus no micronutrient treatment, and colostrum treatment versus no colostrum treatment.

**Results:**

Overall, consumption of the micronutrient mix significantly enhanced delayed-type hypersensitivity (DTH) responses (p < 0.05). Adjusted covariance analysis showed a positive association between DTH and age. Separate analysis of younger and older age groups indicated that it was the older population that benefited from micronutrient consumption. The other immune function parameters including responses to systemic tetanus and oral typhoid vaccination, phagocytosis, oxidative burst, lymphocyte proliferation and lymphocyte subset distribution were neither affected by the consumption of micronutrients nor by the consumption of bovine colostrum concentrate.

**Conclusion:**

Consumption of bovine colostrum had no effect on any of the immune parameters assessed. The micronutrient mix enhanced cellular immunity as measured by DTH, with an increased effect by incremental age, but did not affect any of the other immune parameters measured. Although correlations between decreased DTH and enhanced risk of certain infection have been reported, it remains unclear whether and enhanced DTH response actually improves immune defense. The present data suggests that improvement of immune parameters in a population with a generally good immune and nutritional status is limited and that improvement of immune function in this population may be difficult.

## Background

Supplementation of nutritional deficiencies helps to restore immune function and contributes to increased resistance to infections in malnourished subjects [1]. The literature is less clear however, on the suggested benefits of dietary supplementation for immune function in healthy well nourished subjects without overt signs of compromised immune function. Still, there is strong interest among consumers for food products that can help optimize their immune system and numerous products claiming this, are on the market. Among the food constituents frequently associated with beneficial effects on immune function are micronutrients such as vitamin C, vitamin E, β-carotene and zinc, and also the first milk produced after parturition (colostrum).

Studies suggest that vitamin C may enhance immune functions such as phagocytosis, neutrophil chemotaxis and lymphocyte proliferation [2-4], but contrasting findings have also been published [5-7].

Vitamin E has been shown to enhance antibody responses to hepatitis B- and tetanus vaccination [8] and to increase delayed type hypersensitivity (DTH) response in healthy elderly people [8,9], β-carotene can affect immune function after conversion to vitamin A, but also influences immune activity beyond its pro-vitamin A activity [10]. The most consistent findings of β-carotene activity on immune function are enhanced NK cell activity and TNF-α production [11-13]. Supplementation with 15–25 mg zinc per day in elderly people showed no improvement of the DTH response, lymphocyte proliferation or NK cell activity [14-16]. Higher doses (> 100 mg/day) may improve certain immune aspects in elderly, although some findings are controversial [14,17,18].

Colostrum contains high concentrations of natural immunoglobulins (mainly IgG) that can bind and neutralize pathogens in the intestinal tract, bactericidal factors such as lactoferrin, lactoperoxidase and lysozyme, and growth factors and cytokines that may improve immune defense and gut-barrier function [19-21]. However, data to substantiate suggested benefits of natural colostrum are limited. Natural bovine colostrum has been shown to stimulate *in vitro *phagocytic activity of leukocytes from cattle [22] and human [23]. Consumption of 100 ml colostrum daily for 7 days by volunteers that were orally vaccinated to S.typhi, resulted in more volunteers with a high number of specific IgA secreting cells, although the average number of specific antibody secreting cells was not increased [24].

The current study was designed to investigate the efficacy of commercially viable- and "realistically-to-consume amounts" of bovine colostrum concentrate (Proventra^®^) and a mix of micronutrients (vitamins E & C, β-carotene and zinc) on immune function in healthy immuno-competent subjects aged 40–80 y. Using a complete 2*2 factorial design, subjects were supplemented with bovine colostrum, a mixture of the micronutrients, a combination of bovine colostrum and micronutrients, or a placebo, for 10 weeks. A range of immunological markers was measured, which were identified as relevant for assessing immune function and comprised aspects of innate and adaptive immune function [25]. These included the response to tetanus and (oral) typhoid vaccination, delayed-type hypersensitivity (DTH) responses, phagocytosis, oxidative burst, lymphocyte proliferation and lymphocyte subset distribution.

## Methods

### Subjects and study design

This study was conducted from September to December 2001 at the Unilever Food & Health Research Institute, Vlaardingen, The Netherlands, after approval by the Medical Ethical Committee of Unilever R&D Vlaardingen. At an oral briefing 394 interested candidates completed a questionnaire, resulting in 110 rejections based on exclusion criteria and 21 withdrawals. Two hundred and sixty-three subjects were invited for physical examination. One hundred seventy seven subjects were found eligible. Two subjects withdrew and 35 were excluded by lot. The remaining 140 subjects were randomized to the treatments. Three subjects withdrew after randomization but before the start of the study. One subject was replaced. Finally, one hundred and thirty-eight subjects entered the study. Subjects were healthy, non-smoking, males and females 40–80 y of age, with average Dutch dietary habits, BMI ≥ 18,0 < 32,0 kg/m^2^, normal clinical chemistry, tetanus antibody titers < 700 IU/L, and plasma vitamin E levels ≤ 40 μmol/L. Exclusion criteria were: known diseases, medication affecting immune function and/or intestinal flora, lactose intolerance, high IgE titers to bovine milk or prior typhus vaccination. Subjects were allocated to one of four treatment groups, stratified for gender and randomized for age, basal plasma vitamin E concentrations and titer of tetanus antibodies.

Using a complete 2*2 factorial design, subjects received: (1) control product (2) product containing 1.2 g/d bovine colostrum concentrate (equivalent to ~430 mg IgG), (3) a product containing a mix of micronutrients or (4) a product containing both the bovine colostrum concentrate and the micronutrient mix. The active ingredients were supplied in single serving sachets containing powdered drinks based on skim milk powder, sugar and maltrodextrin. Other ingredients were citric acid, lecithin, colorant and flavoring. The levels of active ingredients are listed in Table 1. Bovine colostrum concentrate (Proventra^®^) was obtained from GalaGen Inc.(Minnetonka MN) as a spray dried sterile filterable powder from the whey fraction of non-specific bovine colostrum. The formulation was developed at the Unilever Health Institute and produced at Budelpack Hamont N.V./S.A. (Hamont-Achel, Belgium). Participants were asked to consume one serving of water-soluble powder dissolved in a glass of water (100 ml) at breakfast and one at dinner. The control and test products were supplied to the participants (pre-portioned in 20 g amounts in two flavors) every 2 weeks. Compliance to dietary restrictions and test product consumption was evaluated at 2 week intervals using a questionnaire.

**Table 1:** Composition of study products^1^. Content of the study products was determined at baseline and for micronutrients E and C and β-carotene also at week 6 and week 10. Of each group, both mango- and orange flavored products were analyzed. Data are presented as the contents of 2 packages, which represents a daily dose.

		**Control**		**Colostrum**		**Micronutrients**		**Colostrum + Micronutrients**	
	**target**	**orange**	**mango**	**orange**	**mango**	**orange**	**mango**	**orange**	**mango**
Energy* (kJ)		628	627	626	625	625	624	623	622
Moisture (%)*		2.6 ± 0.1	2.25 ± 0.25	2.65 ± 0.15	3.15 ± 0.25	2.3 ± 0.1	2.5 ± 0.2	3.1 ± 0.1	3.1 ± 0.1
Fat (%)*		5.5 ± 0.1	5.45 ± 0.05	5.5 ± 0.1	5.9 ± 0.3	6.2 ± 0	6.4 ± 0.1	6.6 v 0.2	6.2 ± 0
Protein (%)*		17.3 ± 0.1	17.6 ± 0.1	16.9 ± 0.1	17.1 ± 0.25	17.1 ± 0.2	16.9 ± 0.15	16.9 ± 0.15	17.2 ± 0.3
									
IgG (mg)*^2^		-	-	422	466	-	-	430	416
									
Vitamin E (mg)									
Baseline	200	-	-	-	-	333 ± 8	314 ± 1.4	296 ± 8.8	293 ± 1
week 6		-	-	-	-	272 ± 2.7	278 ± 4.2	268 ± 0.5	264 ± 0.3
week 10		-	-	-	-	281 ± 0.3	279 ± 2.8	290 ± 0.8	286 ± 0.2
β-carotene (mg)									
Baseline	10	-	-	-	-	12.6 ± 0.2	13.2 ± 0.5	12.3 ± 0.4	12.2 ± 0.4
week 6		-	-	-	-	11.0 ± 0	10.8 ± 0.2	11.2 ± 0,2	10.8 ± 0.4
week 10		-	-	-	-	12.7 ± 0.4	12.9 ± 0.1	12.6 ± 0.2	12.1 ± 0.2
Vitamin C (mg)									
Baseline	250	-	-	-	-	365 ± 8	351 ± 6	369 ± 6	385 ± 10
week 6		-	-	-	-	395 ± 15	381 ± 4	364 ± 17	345 ± 27
week 10		-	-	-	-	382 ± 6	386 ± 4	382 ± 5	390 ± 3
									
Zinc (mg) *	15	-	-	-	-	14.6 ± 2.04	15.3 ± 1.83	15.09 ± 0.3	16.04 ± 1.28

* parameters identified by * were determined at baseline only. - not determined.

^1 ^data are expressed as mean ± SD, except for (calculated) energy and IgG, which are given as means.

^2^IgG was determined by radioimmunodiffusion by Galagen Inc. Mean concentrations of 2 tests were reported without SD.

Participants were instructed to maintain their habitual diet and refrain from using supplements containing vitamin E, vitamin C, β-carotene, zinc, pre- and probiotics and supplements claiming to affect immune function or gut health. Before the intervention, baseline measures were taken of plasma micronutrient concentrations, DTH response, vaccine-specific antibodies and *ex vivo *immune functions (leukocyte subsets, phagocytosis, oxidative burst, lymphocyte proliferation, cytokine production). These measures were repeated after 6 weeks intervention. Subsequently, subjects received an intra-muscular tetanus vaccine and an oral typhoid vaccine. The dietary intervention was continued until week 10 and specific responses to the vaccines were measured in week 8 and 10. In addition, a final measurement of the plasma concentrations of vitamin E, and C, and β-carotene was made at week 10.

### Blood and saliva sampling

Fasted blood samples were obtained at baseline, week 6, week 8 and week 10 by venipuncture. Aliquots of plasma and serum were stored at -70°C until further analysis. Plasma for vitamin C analysis was mixed with metaphosphoric acid and stored at -20°C. Saliva stimulated by chewing on parafilm was collected at baseline, week 8 and week 10. Samples were kept on ice, centrifuged 15 minutes at 3000 g at 4°C to pellet debris. Supernatants were aliquoted and stored at -70°C until further analysis.

### Vaccination

After 6 weeks, subjects were vaccinated for tetanus (single i.m. injection of tetanus toxoid vaccine, Te Anatoxal Berna, Primmed BV, Almere, NL) and typhoid (three oral doses on alternate days of Vivotif Berna, Primmed BV, Almere, NL), to assess both systemic and mucosal immune responses. Vaccine-specific antibodies were determined at baseline and weeks 8 and 10 (i.e. 2 and 4 weeks after vaccination). Proliferative responses to the vaccine antigen were determined at baseline and week 10. Tetanus specific antibody titers were determined by ELISA (Tetanus toxoid Sensitive IgG antibody test, Gamma Angleur-Liege, Belgium) according to manufacturer's instructions. Subjects with ≥ 4-fold increase over baseline were classified as responders to the tetanus vaccination. Antibodies to S.typhi Ty21a LPS were determined by ELISA at the Berna Swiss Serum and Vaccine Institute, Berne, Switzerland according to a standard operating procedure. Subjects with ≥ 2-fold increase over baseline for postvaccination titers > 1 E.U/ml, or ≥ 3-fold increase over baseline for postvaccination titers < 1 E.U/ml were classified as responders [26] (Dr.J. Que, Swiss Vaccine Institute, personal communication)

### Delayed-type hypersensitivity (DTH) response

Delayed-type recall responses were assessed at baseline and after 6 weeks intervention using the Multitest CMI applicator from Pasteur Mérieux (Lyon, France) for simultaneous intra-cutaneous application of six antigens (Tetanus, Diphteria, Streptococcus group C, Candida albicans, Trichophyton mentagrophytes, Proteus mirabilis). Tuberculin was removed from the applicator to avoid interference with tuberculosis surveillance. Forty-eight ± 3 hours after application, the number of indurations and induration diameters were recorded. The DTH response of each subject was calculated as the number of positive (> 2 mm) responses out of the six test antigens and the sum of diameters of all positive responses.

### Plasma levels of micronutrients

Micronutrient concentrations were determined in plasma samples collected at baseline, week 6 and week 10. α-Tocopherol and β-carotene levels were determined by HPLC, performed at Analytico Medinet B.V (Breda, Netherlands) according to standard procedures. Vitamin C was determined enzymatically on EDTA plasma samples (stored frozen with 4.5 % MPA) according to Vuilleumier and Keck (1989) adapted for use on a Packard Multiprobe ll HT analyzer.

### Leukocyte subsets

Leukocyte subsets were determined in heparinized whole blood at baseline, week 6 and week 10 using a standardized four-color flowcytometric method (TetraCHROME, Beckman Coulter Nederland BV, Mijdrecht with added CD16-RD1 from Immunotech). Absolute numbers and percentages of leukocytes (CD45+), T cells (CD45+/CD3+), helper T cells (CD3+/CD4+/CD45+), cytotoxic/suppressor T cells (CD3+/CD8+/CD45+), precursor B cells and B cells (CD19+/CD45+) and NK cells (CD3-/(CD56/CD16)+/CD45+) were determined. Samples were analyzed on the Coulter EPICS XL flowcytometer (Beckman Coulter Nederland BV, Mijdrecht) using fully automated tetraONE SYSTEM software (Beckman Coulter Nederland BV, Mijdrecht).

### Phagocytosis and oxidative burst

Phagocytic activity and oxidative burst were determined in heparinized whole blood at baseline and week 6. For phagocytosis, 50 ul of whole blood was incubated at 37°C for 5 minutes with FITC-labeled *E.coli *(Phagotest^®^, Orpegen, Heidelberg, Germany), or for 10 minutes with unlabeled *E.coli *(Phagoburst^®^, Orpegen, Heidelberg, Germany) according to the manufacturers instructions. Control samples were incubated with bacteria on ice. For oxidative burst, samples were subsequently incubated with dihydrorhodamine 123 substrate for 10 minutes. For both assays erythrocytes were lysed and samples were fixed and stained with propidium iodide. Analysis was performed by flowcytometry (Coulter EPICS XL, Beckman Coulter Nederland BV, Mijdrecht). Leukocytes were gated into monocyte and granulocyte populations according to FSC/SSC profile. The percentage of cells responding with phagocytosis/oxidative burst in these populations and the mean fluorescence of these cells were determined.

### Lymphocyte proliferation

Mitogen-induced lymphocyte proliferation was assessed in 10* diluted whole blood cultures at baseline and week 6. Samples were incubated in quadruplicate with a mixture of anti-CD3 and anti-CD28 monoclonal antibodies (PeliCluster, Sanquin Reagents, Amsterdam, the Netherlands) at final concentrations of 0, 0.1 and 1 μg/ml.

Antigen-specific proliferation was assessed in PBMC cultures (1.10^6 ^PBMC/ml in 10 % autologous plasma) at baseline and week 10. Cells were incubated in quadruplicate cultures with tetanus toxoid (NIBSC, Hertfordshire, UK) at final concentrations of 0, 1, 2.5 and 5 lfu/ml or with heat-inactivated S.typhi Ty21a cells at 0, 0.1*10^6^, 1*10^6 ^and 5*10^6 ^cells/ml. Bacterial cells were cultured from the oral vaccine capsule (Vivotif Berna, Primmed BV, Almere, NL) by resuspending the capsule contents in sterile water and plating onto BHI agar plates for single colony isolation. An isolated colony was then expanded in Luria broth, heat inactivated and stored at 4°C until use. Desired *S.typhi *Ty21a concentrations were prepared in RPMI culture medium and incubated with PBMC.

Cultures were incubated for 3 (mitogenic) or 6 days (antigen specific) at 37°C, 5% CO_2_. Eighteen hours before the end of the incubation periods, 0.4 μCi methyl-^3^H-thymidine (Amersham Pharmacia, Buckinghamshire, UK) was added per well. Incorporated ^3^H-thymidine was measured in a β-scintillationcounter (Microbeta Trilux scintillationcounter, Wallac, Turku, Finland).

### Statistics

The power of the study was based on a study by Meydani et al [8]. In this study 200 mg d, l-α-tocopherol increased the antibody response to tetanus toxoid vaccine 1.7-fold (*P *= 0.04). From these data the variance of the natural log was calculated to be 0.8. It is estimated that a difference of 1.5 would indicate a significant increase in antibody titers. In a 2 × 2 factorial design (with A and B as factors), 35 volunteers per group (70 per level of A or B) would be sufficient to detect a 1.53-fold increase in response to tetanus toxoid vaccine (power of 0.8, alpha = 0.05). Therefore, a total of 140 subjects were recruited.

Data were analyzed by analysis of variance with colostrum treatment, micronutrient treatment, cohorts and gender as factors. As in none of the tested parameters a significant interaction between colostrum and micronutrient treatment was found, groups were combined to test micronutrient treatment versus no micronutrient treatment, and colostrum treatment versus no colostrum treatment. Occasionally individual datapoints were missing due to laboratory error or missing samples. The correct numbers on which statistical analyses were based are provided with the corresponding datasets.

Results are presented as least square means (LSM) with 95% confidence interval (95% CI) for the four treatment groups. The p-values of the statistical analyses are based on the combination of the groups (2*2 factorial design). A p-value < 0.05 was considered significant. The distributions of the response to vaccination (TT IgG and Ty21a IgG and IgA) were skewed, therefore, natural logarithm-transformed values for these variables were used for statistical testing. The delta compared to the baseline values of these parameters was expressed as a factor.

Covariance-analysis, adjusted for age, gender, treatment, cohort and interaction terms between age and treatments was used to evaluate the association between age and immune parameters. To show the effect of age, we subdivided the group in two age groups, using the median age of 56 years as cut-off point. All analysis were performed using the SAS statistical package version 8.2 (SAS institute, NC, US).

## Results

### Composition of test products

The average amounts of active ingredients were comparable between the individual batches, were higher than the target levels and remained stable over the time course of the study (Table 1).

### Baseline characteristics, randomization, and compliance

Four subjects dropped out of the study for reasons unrelated to the intervention. One of these subjects dropped out after 6 weeks, still leaving a complete data set except for the vaccination-related measurements. During a blind review 4 more volunteers were excluded from the analyses due to body weight loss > 10 kg during the study, number of B-cells vastly exceeding reference range, non-compliance and illness, respectively.

Characteristics of the 131 subjects included in the analysis are given in Table 2. Average BMI, age, vitamin E concentrations and response to tetanus vaccination were similar in all groups. Of the subjects completing the study 68% were women and the average age was 57 ± 10 years. The overall compliance was good, 92% of the volunteers consumed more than 98 % of the supplied products. One person did not consume 14 packages of the total of 140. This person was removed from the dataset in the blind review.

**Table 2:** Baseline characteristics of subjects who completed the study

	**Total**	**Control**	**Colostrum**	**Micronutrients**	**Micronutrients + colostrum**
Gender, n					
Total	131	31	33	32	35
Men	42	10	11	10	11
Women	89	21	22	22	24
Age, years					
Total	56.7 ± 10.0	56.0 ± 10.1	56.8 ± 10.2	57.5 ± 10.1	56.3 ± 9.9
Men	61.4 ± 10.1	57.7 ± 9.9	62.4 ± 10.9	61.9 ± 11.4	63.4 ± 8.5
Women	54.4 ± 9.2	55.2 ± 10.3	54.1 ± 8.9	55.5 ± 9.1	53.0 ± 8.8
BMI, kg/m^2^					
Total	25.3 ± 2.7	25.1 ± 3.2	25.4 ± 2.50	24.9 ± 2.5	25.6 ± 2.5
Men	26.0 ± 2.6	26.5 ± 3.4	26.0 ± 2.3	26.0 ± 2.3	25.5 ± 2.5
Women	24.9 ± 2.7	24.5 ± 3.1	25.1 ± 2.6	24.3 ± 2.5	25.6 ± 2.5
Vitamin E, μmol/L					
Total	27.1 ± 7.2	27.3 ± 10.0	27.9 ± 6.3	26.9 ± 4.5	26.4 ± 7.2
Men	28.1 ± 9.7	33.6 ± 13.7	24.5 ± 6.6	25.8 ± 3.8	28.9 ± 10.5
Women	26.6 ± 5.6	24.3 ± 6.0	29.6 ± 5.6	27.4 ± 4.7	25.3 ± 4.9
Tetanus titers, IU/L					
Total^1^	-	126 (90.2 – 175)	154 (111 – 212)	147 (105 – 206)	138 (101 – 189)

Data are represented as mean ± SD

^1 ^Tetanus titers were determined at the screening examination, mean (95% CI)

### Plasma concentrations of micronutrients

**Figure 1:**
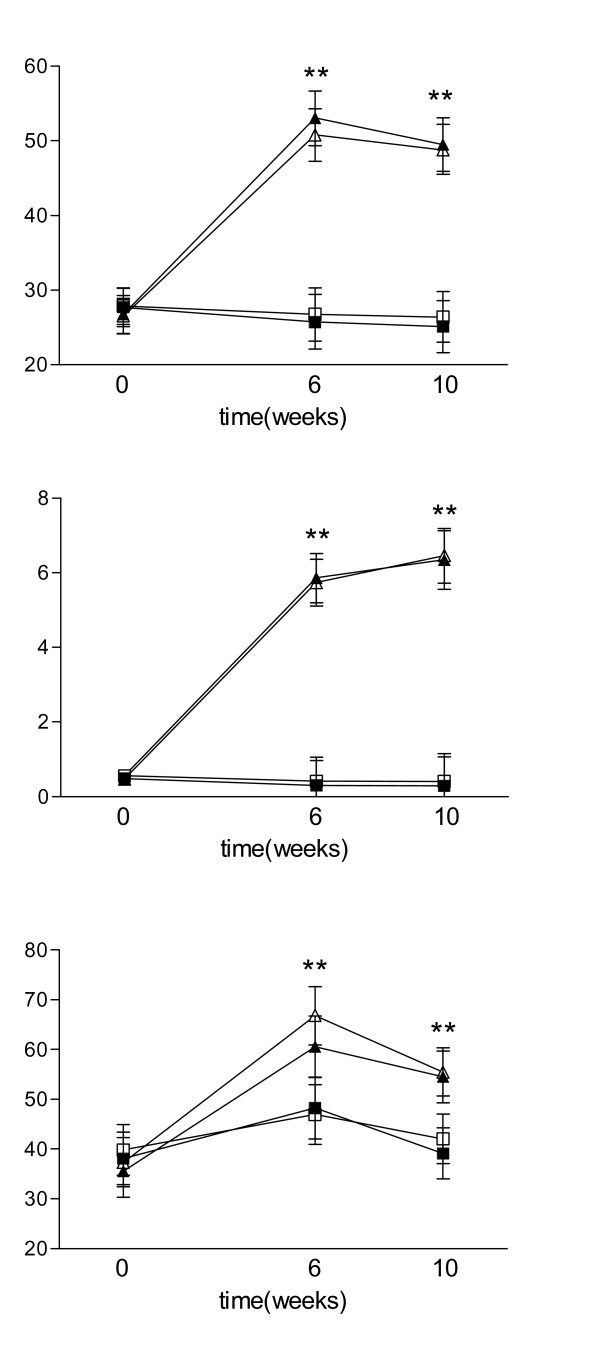
**Plasma concentrations of vitamin C & E and β-carotene**. Plasma concentrations of vitamin C & E and β-carotene were measured at base line, 6 and 10 weeks. Data are expressed as LSM (± 95% CI). Levels in the micronutrient groups (micronutrient group ▲ and micronutrient + colostrum group △) are significantly different from levels in non-vitamin group (control ■ and colostrum group □) with ** p < 0.0001.

At baseline, plasma concentrations of vitamin E & C and β-carotene were comparable between the groups, i.e. ~27 μmol/L for α-tocopherol, ~0.5 μmol/L for β-carotene and ~37 μmol/L for vitamin C. At week 6, α-tocopherol concentrations had increased by more than 90 %, vitamin C by more than 70 % and β-carotene over 900 % (to ~5.7 μmol/L) in the groups consuming the micronutrient containing drinks (P < 0.001 for all), whereas no change was observed in the two non-micronutrient groups (Figure 1). All concentrations seemed to plateau by week 6 as no further increase was observed after 10 weeks intervention. Simultaneous consumption of colostrum did not affect the concentrations of these micronutrients in plasma.

### Response to vaccination

As expected, tetanus-specific IgG increased markedly after vaccination. Compared to baseline, titers were 17-fold increased in the control group, 23-fold in micronutrient group, 27-fold in colostrum group and 37-fold in micronutrient + colostrum group 2 weeks after vaccination (week 8 of the intervention) (Table 3). However, none of the treatments significantly affected the mean titers, the percentage of vaccine-responders (81%, 91%, 94% and 97% at week 10 for control, colostrum, micronutrient, micronutrient + colostrum, respectively), or the mean titers of the responders (data not shown).

**Table 3:** response to tetanus and typoid vaccination

	**Control (n = 31)**	**Colostrum (n = 33)**	**Micronutrients (n = 31)**	**Colostrum+Micronutrients (n = 35)^2^**
*Tetanus toxoid-specific IgG levels in serum (IU/L)*				
Baseline	126 (90.2 – 175)	154 (111 – 212)	147 (105 – 206)	138 (101 – 189)
Week 8	2141 (1040 – 4407)	4074 (2024 – 8200)	3368 (1625 – 6982)	5131 (2579 – 10208)
Week 10	3138 (1713 – 5748)	4728 (2629 – 8500)	4206 (2282 – 7753)	5330 (3013 – 9431)
*S. typhi Ty21a LPS-specific IgG in serum (E.U/mL)*				
Baseline^3^	3.37 (2.24 – 5.07)	3.93 (2.64 – 5.84)	5.39 (3.54 – 8.20)	6.48 (4.40 – 9.52)
Week 8^3^	12.6 (9.02 – 17.6)	16.5 (12.0 – 22.8)	19.5 (13.9 – 27.5)	21.6 (15.7 – 29.6)
Week 10^3^	10.7 (7.67 – 14.8)	14.0 (10.1 – 19.2)	16.7 (11.9 – 23.4)	18.4 (13.5 – 25.2)
*S. typhi Ty21a LPS-specific IgA in serum (E.U./mL)*				
Baseline	1.21 (0.85 – 1.72)	1.07 (0.76 – 1.51)	1.36 (0.95 – 1.95)	1.43 (1.03 – 1.99)
Week 8	2.70 (1.89 – 3.86)	3.40 (2.40 – 4.81)	3.49 (2.42 – 5.04)	3.73 (2.65 – 5.24)
Week 10	1.80 (1.29 – 2.52)	1.86 (1.35 – 2.57)	2.24 (1.59 – 3.14)	2.20 (1.60 – 3.01)

^1 ^values are expressed as lsmeans (95% CI).

^2 ^n = 34 in serum at week 8, n = 33 for saliva at week 8.

^3^Micronutrient group (Micronutrients group and Colostrum + Micronutrients group) is significantly different from non-micronutrient group (Control group and Colostrum group) (P < 0.05).

After the oral typhus vaccination, specific IgG and IgA titers in serum were increased in all groups 2 weeks after vaccination (week 8 of the intervention) after which titers tended to decrease by 4 weeks after vaccination (week 10 of the intervention). The specific IgG antibody titers were significantly higher in the micronutrient groups compared to the non-micronutrient groups, but this significant difference was already present at baseline and could therefore not be attributed to the treatments (Table 3). Increase compared to baseline values was comparable in all groups and treatments did also not significantly affect the percentage of responders (74%, 75%, 60% and 70% for IgG and 42%, 64%, 53% and 59% for IgA at 2 weeks after vaccination for control, colostrum, micronutrient, micronutrient + colostrum, respectively), or the mean titer of the responders (data not shown). Typhus-specific salivary IgA was only found in very few subjects and only 4 out of the 129 tested individuals could be marked as responder based on salivary IgA responses (data not shown).

### DTH-response

The DTH response measured as the number of positive indurations increased in all groups. In the micronutrient group the average number (+CI) increased from 1.05 (0.72 – 1.38) to 1.73 (1.28 – 2.18) at week 6, in the micronutrient +colostrum group from 1.11 (0.80 – 1.42) to 1.62 (1.20 – 2.04), in the colostrum group from 1.00 (0.68 – 1.31) to 1.33 (0.91 – 1.76) and in the control group from 0.73 (0.39 – 1.06) to 0.85 (0.39 – 1.30). Both the absolute values at week 6 as well as the increase compared to baseline, were significantly different between the micronutrient and the non-micronutrient groups (p = 0.009 and p = 0.031, respectively).

The cumulative induration diameter tended to increase in all groups although neither the absolute values at week 6 nor the increases compared to baseline were significantly different between the micronutrient and the non-micronutrient groups (p = 0.068 and p = 0.16 respectively).

**Figure 2:**
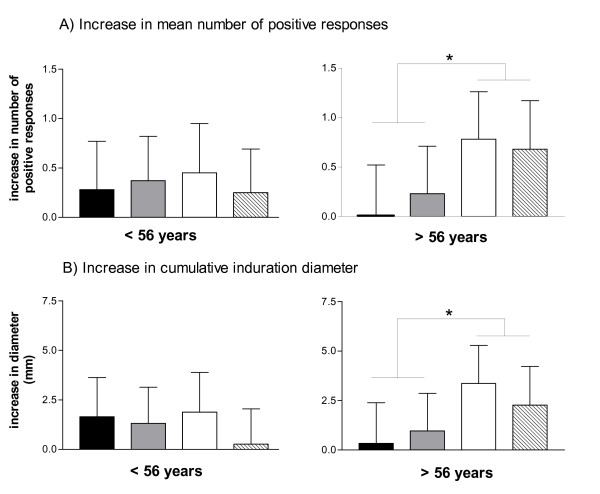
**Delayed-type hypersensitivity response in two age groups**. DTH responses were assessed using the CMI multitest at baseline and at 6 weeks. Because of significant interaction between age and treatment group (p < 0.05), the population was split around the median age of 56 y in younger (< 56 y) and older (> 56 y) subjects. Average age of the younger subjects was 48 ± 4 y, with a BMI of 24.5 ± 2.8, comprising 12 men and 52 women. The average age of the older subjects was 65 ± 6 y, with a BMI of 26.0 ± 2.3, comprising of 30 men and 36 women. Both age categories were evenly spread over the treatments with 15–19 subjects per treatment per age category. Data are reported as LSM of the mean increase in the number of positive responses (A) and increase of the cumulative diameter of the positive responses (B). Responses in the micronutrient groups (micronutrient (white) and micronutrient + colostrum group (hatched)) are significantly different from levels in non-vitamin group (control (black) and colostrum group (grey)) with * p < 0.05.

Covariance-analysis, adjusted for age, gender, cohort, treatment and interaction terms between age and treatment, indicated a positive association between DTH response and age. To show this association, we subdivided the group in two age groups, using the median age of 56 years as cut-off point. Both the number of positive indurations and the cumulative induration diameter were significantly different between the micronutrient group and the non-micronutrient group in the older age group with p < 0.05 but not in the younger (see Figure 2).

### Leukocyte subsets

A small but significant (p < 0.05) decrease in the percentage of B cells was observed at 10 weeks in the micronutrient-groups compared to the non-micronutrient groups (LSM (CI) for control, colostrum, micronutrient, micronutrient + colostrum were 14.6 (12.9–16.4), 13.2 (11.5–15.0), 11.8 (10.0–13.6), 12.1(10.4–13.8), respectively). As there were no related observations made, the biological relevance of this observation is unclear and we therefore regard this as an incidental finding. Changes in all other leukocyte populations, i.e. total leukocytes, T cells, helper T cells, cytotoxic/suppressor T cells, NK cells and NK-T cells were limited and did not differ between groups, either in absolute numbers or percentages (data not shown). The observed values were well within normal ranges.

### Phagocytosis and oxidative burst

Consumption of colostrum and/or micronutrient-containing test products did not affect the percentage of phagocyting granulocytes when whole blood taken from these subjects was *ex vivo *stimulated with non-opsonised or opsonised *E coli*, nor did it affect phagocyting activity per cell as indicated by the mean fluorescence (Table 4). Retrospective power calculation indicated that the study had sufficient power to allow detection of changes in phagocyting granulocytes of > 4 %. The oxidative burst in the phagocyte population as elicited by an *E. coli *-challenge, was also not affected (data not shown).

**Table 4:** phagocytosis of opsonized E coli

	Control (n = 31)	Colostrum (n = 33)	Micronutrients (n = 31)	Colostrum + Micronutrients (n = 35)
Granulocytes (% of leukocytes)				
Baseline	47.9 (44.5 – 51.2)	49.1 (45.8 – 52.3)	49.7 (46.3 – 53.0)	52.4 (49.3 – 55.6)
Week 6	49.5 (46.3 – 52.7)	49.5 (46.4 – 52.6)	50.0 (46.8 – 53.3)	50.4 (47.3 – 53.4)
*Difference week 0–6*	*1.63 (-1.4 – 4.71)*	*0.45 (-2.5 – 3.43)*	*0.39 (-2.7 – 3.49)*	*-2.1 (-4.9 – 0.84)*
Positive granulocytes (% of total granulocytes)				
Baseline	65.6 (62.5 – 68.6)	63.1 (60.2 – 66.0)	64.9 (61.8 – 67.9)	65.2 (62.4 – 68.1)
Week 6	60.4 (57.2 – 63.6)	58.4 (55.3 – 61.5)	56.7(53.4 – 59.9)	58.0 (55.0 – 61.0)
*Difference week 0–6*	*-5.1 (-8.7 – -1.6)*	*-4.7 (-8.2 – -1.2)*	*-8.2 (-12 – -4.6)*	*-7.2 (-11 – -3.8)*
Granulocytes fluorescence mean				
Baseline	422 (393 – 451)	390 (362 – 418)	410 (381 – 439)	408 (381 – 435)
Week 6	450 (423 – 478)	438 (411 – 465)	407 (379 – 436)	430 (404 – 456)
*Difference week 0–6*	*28.7 (-5.8 – 63.2)*	*47.9 (14.5 – 81.3)*	*-2.6 (-37 – 32.3)*	*21.6 (-11 – 54.1)*

^1 ^values are expressed as lsmeans (95% CI)

### Lymphocyte proliferation

Whole blood cultures stimulated with the mitogenic stimulus anti-CD3/CD28 demonstrated the expected relationship between stimulus concentration and proliferative response. A few significant differences were observed between the groups, but these were already apparent at baseline and the treatments did not affect the mitogenic proliferative responses as assessed at week 6 (data not shown). Stimulation of PBMC cultures with tetanus toxoid indicated the expected increase in responsiveness following the tetanus vaccination (at 4 weeks after vaccination compared to baseline), but the responses were not affected by the treatments (not shown). In contrast, proliferation induced by *S.typhi *Ty21a cells did not increase after vaccination. Already at baseline, proliferative responses increased with increasing concentration of *S.typhi *Ty21a cells, but these responses did not increase after typhoid vaccination and were also not affected by the treatments (not shown).

## Discussion

Out of the range of immune function parameters examined, only the DTH response was positively affected by micronutrient intervention, whereas none of the immune parameters were affected by consumption of bovine colostrum. Separate analyses of older versus younger subjects, demonstrated that both DTH parameters, i.e. number of positive responses and diameter of indurations, were significantly enhanced in the older population, whereas this effect was not observed in the younger population.

The evidence for a positive effect of micronutrient supplementation on DTH seems to be consistent. Other studies have demonstrated an increase in the DTH response of elderly after micronutrient supplementation, especially by vitamin E [8,9]. Also β-carotene, although in higher doses, has been described to have stimulating effects on DTH [27].

How and if an enhanced DTH response relates to improved resistance remains to be elucidated. Studies suggest a correlation between a low DTH score and risk of e.g. diarrhoeal disease in Kenyan children [28] septic-related deaths in surgical patients [29] pneumonia in elderly [30] and progression to AIDS in HIV positive individuals [31]. Interestingly, despite the more or less consistent observation that vitamin E improves the DTH response, conflicting reports were published on the effect of vitamin E on resistance to respiratory infections. One study showed that consumption of 200 mg vitamin E daily for 15 months does not improve resistance to acute respiratory tract infections [32]. In fact, it was shown that the severity of respiratory tract infection was worse in vitamin E supplemented groups, as well as the duration of the symptoms. A second study by Meydani et al [33] reported a protective effect of 200 IU daily of vitamin E for 1 year on upper respiratory tract infections, especially the common cold. How these observations correlate with respect to enhanced DTH after vitamin E suppletion remains elusive. Accumulation of vitamin E in tissues may account for detrimental effects of vitamin E and vitamin-E associated enhanced immune reactivity could either account for worsening of symptoms or improvement of resistance to certain infections. Alternatively, the DTH reaction may be an immunological parameter that is not associated with resistance to respiratory infections.

The absence of positive effects of the micronutrient mix on other immune parameters or of the colostrum preparation on all parameters examined, may largely be explained by the generally good health and nutritional status of the subjects. This is illustrated by the fact that successful immune enhancement is generally achieved in populations with suboptimal immune function such as elderly and athletes [34]. Interestingly, in elderly that retain a good nutritional status, decline in immne fucntion is not apparent [35,36].

In our study population the average baseline concentrations of plasma vitamin E, vitamin C and β-carotene as well as the post-intervention vitamin E and C levels were comparable to what has been described in other studies [8,11,27,32,37] and are indicative for a generally well-nourished population. The observed 900% rise in plasma β-carotene, which is higher than reported increases of 300–600% in other studies using similar doses [11,37,38], may be explained by the fact that the test products contained the emulsifier lecithin in a molar ratio of ~70 relative to ß-carotene. Emulsifiers and fat from the formulation can help form micelles, which are required for absorption of carotenoids during lipolysis, thereby improving the bioavailabilty of lipophilic molecules such as ß-carotene [39-42].

Also in the older subpopulation a lower nutritional status can not explain the observed benefit, since baseline micronutrient concentrations were higher (vitamin E) or similar (vitamin C and ∃-carotene) in the subjects older than 56 years compared to the younger subjects (29.1 ± 7.6 umol/L vs 25.1 ± 6.2 umol/L, P = 0.0012 for vitamin E). Similarly, the size of the increase in micronutrient levels did not seem to correlate with the effect on the DTH since increases were similar in both populations.

Despite a few publications indicating the stimulatory effect of colostrum on phagocytic activity in vitro [22,23,43], which we confirmed with human leukocytes (data not shown), daily consumption of 1.2 g of bovine colostrum concentrate by volunteers in the current study did not affect ex-vivo phagocytic activity. Similarly, none of the other investigated immune parameters were significantly influenced by colostrum consumption, although the response to tetanus vaccination tended to be somewhat higher (p = 0.14).

Three studies have reported effects on immune function and resistance to infections after consumption of natural colostrum (enhanced response to oral typhoid vaccine [24], reduced number of URTI symptoms [44], and increased salivary IgA [45]). However, the doses of colostrum used in these studies were up to 60 g/day. These high doses may be feasible in the context of severe physical exercise for which some of these studies were designed or for clinical nutrition, but for daily consumption by healthy consumers only lower doses would be economically feasible.

## Conclusion

Low daily doses of bovine colostrum do not improve a series of immune function parameters in healthy volunteers. Micronutrient suppletion of vitamins E & C, ß-carotene and zinc positively affect the DTH response, especially in elderly subjects. How this may relate to improved resistance remains to be elucidated. Improvement of other immune parameters in individuals with a generally good immune and nutritional status may be difficult and it may take individuals with (moderately) impaired immune function to benefit from this type of nutritional intervention.

## Competing interests

All authors are employed by Unilever who financed the study and publication.

## Authors' contributions

DW, ML, RvdW and RA contributed to the study design. DW and ML were responsible for the human intervention study. DW, WH and RA supervised the assays and analyzed the data. DW and WH drafted the initial manuscript and DW and RA edited the final manuscript with contributions of all the authors. All authors read and approved the final manuscript.
